# Laccase Production from *Agrocybe pediades*: Purification and Functional Characterization of a Consistent Laccase Isoenzyme in Liquid Culture

**DOI:** 10.3390/microorganisms11030568

**Published:** 2023-02-24

**Authors:** Paulina González-González, Saúl Gómez-Manzo, Araceli Tomasini, José Luis Martínez y Pérez, Edelmira García Nieto, Arely Anaya-Hernández, Elvia Ortiz Ortiz, Rosa Angélica Castillo Rodríguez, Jaime Marcial-Quino, Alba Mónica Montiel-González

**Affiliations:** 1 Maestría en Ciencias en Sistemas del Ambiente, Centro de Investigación en Genética y Ambiente, Universidad Autónoma de Tlaxcala, Tlaxcala 90120, Mexico; 2 Laboratorio de Bioquímica Genética, Instituto Nacional de Pediatría, Secretaría de Salud, Ciudad de Mexico 04530, Mexico; 3 Departamento de Biotecnología, CBS, Universidad Autónoma Metropolitana-Iztapalapa, Ciudad de Mexico 09340, Mexico; 4 Centro de Investigación en Genética y Ambiente, Universidad Autónoma de Tlaxcala, Tlaxcala 90120, Mexico; 5 Facultad de Odontología, Universidad Autónoma de Tlaxcala, Tlaxcala 90000, Mexico; 6 CICATA Unidad Morelos, Instituto Politécnico Nacional. Boulevard de la Tecnología, 1036 Z-1, P 2/2, Atlacholoaya 62790, Mexico

**Keywords:** *Agrocybe pediades*, extracellular laccase, 2,6-dimethoxyphenol, kinetic parameters

## Abstract

Laccases are valuable enzymes as an excellent ecological alternative for bioremediation issues because they can oxidize persistent xenobiotic compounds. The production and characterization of extracellular laccases from saprotrophic fungi from disturbed environments have been scarcely explored, even though this could diversify their functional characteristics and expand the conditions in which they carry out their catalysis. *Agrocybe pediades*, isolated from a disturbed forest, produces an extracellular laccase in liquid culture. The enzyme was purified, identified and characterized. Copper and hexachlorobenzene do not function as inducers for the laccase produced. Partial amino acid sequences were obtained by LC-MS/MS that share similarity with laccases from other fungi. Purified laccase is a monomer with a molecular mass between 55–60 kDa and had an optimum activity at pH 5.0 and the optimum temperature at 45 °C using 2,6-dimethoxyphenol (2,6-DMP) as substrate. The *K*_m_ and *V*_max_ also determined with 2,6-DMP were 100 μM and 285 μmol∙min^−1^∙mg^−1^, respectively, showing that the laccase of *A*. *pediades* has a higher affinity for this substrate than that of other Agaricales. These features could provide a potential catalyst for different toxic substrates and in the future laccase could be used in environmental recovery processes.

## 1. Introduction

Saprotrophic fungi perform essential ecological functions such as key regulators and decomposers of nutrient cycling in terrestrial ecosystems. These fungi use dead organic matter to degrade plant cell wall components such as polysaccharides (chitin) and polymers (cellulose, hemicellulose, pectin, and lignin) [1,2]. For the processing of these polymers, the production of extracellular enzymes is required, which cause the endo or exoscission of said molecules. Hydrolytic and lignocellulolytic enzymes are responsible for the degradation and mineralization of the components of the cell wall [3]. White rot fungi (WRF), belonging to the basidiomycetes, have the ability to degrade the polymers of lignocellulosic materials [4]. This property is due to the fact that WRF produce large extracellular amounts of lignin-modifying enzymes (LMEs), such as manganese peroxidase (MnP), lignin peroxidase (LiP) and laccase (Lac), which are responsible for the degradation and detoxification of lignocellulosic residues from the environment [4,5,6]. The reactions catalyzed by peroxidases and laccases are very similar, based on the oxidation–reduction process. The action mechanisms of both types of enzymes are through one-electron oxidation, which creates radicals and makes possible the degradation of different phenolic compounds and aromatic amines [5]. However, these enzymes show important differences, for example they differ in their prosthetic group, laccases are enzymes with low redox potential compared to peroxidases, and one of the greatest advantages of laccase is that it uses molecular oxygen (O_2_) as the only co-substrate for its catalysis, unlike peroxidases that require hydrogen peroxide (H_2_O_2_) [6]. Given the ability of LMEs to catalyze organic and inorganic substrates, these enzymes can be used for the treatment of environmental contaminants from agricultural activities, agro-industrial waste, the textile and pharmaceutical industries [7,8]. In reference to this, laccases of fungal origin have received special attention due to their ability to oxidize components of lignin and also various xenobiotic and chemical compounds, including textile dyes [9,10].

Laccases (p-benzenediol:oxygen oxidoreductase, EC 1.10.3.2) are enzymes that are part of the family of proteins known as multicopper blue oxidases and are characterized by containing copper atoms in their active center [11]. In their structure, laccases have three types of copper atoms and one of them is responsible for their characteristic blue color. These enzymes catalyze the transformation of different aromatic and non-aromatic compounds with the concomitant reduction of molecular oxygen (O_2_) to water (H_2_O) [6,12]. The only requirement of molecular oxygen for the catalysis of the compounds has made them usable for different biotechnological applications such as the clarification of beverages, dye degradation and bioremediation of environmental pollutants (chlorophenols, polycyclic aromatic hydrocarbons, pesticides) [9,12].

Laccase activity has been detected in a large number of WRF and it is considered that the main producers of laccases are those belonging to the family of Ascomycetes, Deuteromycetes, although mostly in Basidiomycetes [10,13]. In addition, the purification of extracellular laccases has been reported from fungi such as *Lentinula edodes* [14], *Fusarium solani* [15], *Thermobifida fusca* [16], *Leptosphaerulina chartarum* [17], *Trametes* sp. LAC-01 [18], *T. trogii* [19], *Cerrena unicolor* [20], *Thielavia* sp. [21], *Penicillium chrysogenum* [22] and *Pleurotus eryngii* [23]. The purification of these laccases has allowed us to know more details of their structure. In this context, most fungal laccases have been characterized primarily as monomeric with molecular masses of 55–85 kDa [3] and are highly glycosylated [24]. They have also been reported as homodimeric, heterodimeric, and multimeric [25]. In different WRFs, laccase isoforms have been reported, derived from the presence of more than one gene contained in their genomes, which can be constitutively expressed or induced. Furthermore, experimental data have revealed that the expression of these genes is differential. Like *Pleurotus ostreatus* that contains twelve genes and only some of them are expressed depending on the culture conditions. Goudopoulou et al. [26], when evaluating *P. ostreatus* in oil mill wastewater and carrying out the expression profile, found that only pox3 transcripts were abundantly expressed; while Castanera et al. [27] demonstrated that in liquid and solid cultures, the *lacc2* and *lacc10* genes are mostly expressed over time. It has also been shown that the genes that encode laccases can be positively regulated by factors such as the carbon/nitrogen ratio in the culture or induced by byproduct components (citrus washing pulp) at specific pH and rpm as demonstrated in *P*. *sajor-caju* [28]. Metal ions also influence the expression of laccases during culture [29]. Cu^2+^ has been used to increase laccase production, which has been widely documented in *P. ostreatus* [30,31] and *P. eryngii* [23] species. Similarly, the copper ion showed an increase in the production of laccase during the degradation of the raw vinassa byproduct using *P. sajor-caju* [8]. Mn^2+^ is another metal that regulates the production of these enzymes as recorded in *Coprinus comatus* [32]. Lastly, phenolic or aromatic compounds structurally related to lignin (or derivatives) also have an effect on laccase production [33]. The induction of laccases by this type of compound varies between species of fungi and depends on their structure and concentration. The particular cases, 2,2′-azino-bis(3-ethylbenzothiazoline-6-sulfonic acid) (ABTS) and guaiacol show high induction in the fungus *Cerrena* sp. HYB07 [34]; while 2,5 Xylidine as well as pyrogallol are similar in *Hexagonia hirta* MSF2 [35]. Thus, the diversity of laccases in fungi leads them to perform various physiological functions and present different biochemical properties, particularly their ability to catalyze a large number of substrates [36]. Therefore, it is not trivial that studying the structures and biochemical properties of enzymes from various fungi is a field of current research and growing development because understanding diverse structures and properties enhances new applications of the different resources found.

In the present study, we worked with a fungal strain identified morphologically and molecularly as *Agrocybe pediades*. This fungus is a Basidiomycete that belongs to the family Strophariaceae (order Agaricales) with saprophytic habits, which grows in natural (forest, pastures, on plant remains and soil) or anthropic (gardens, roadsides) areas [37]. Nevertheless, this strain of *A. pediades* was collected from a *Juniperus deppeana* forest in the central region of Mexico, which has been severely affected by different anthropogenic activities, including felling of trees, grazing, crops, use of herbicides and fires. Considering the environmental conditions of development of the fungus, it was of our interest to isolate it and cultivate it in vitro, in order to obtain enzymes with biotechnological potential. In view of this, the fungus was cultivated in liquid media, and it was shown that it produces an extracellular phenoloxidase, which was purified and identified as a laccase. In addition, their biochemical parameters were determined and given the kinetic properties of laccase, this enzyme can be used in future studies for testing on different substrates, including toxic compounds of environmental interest.

## 2. Materials and Methods

### 2.1. Microorganism and Culture Conditions

*A. pediades* strain CIGYA-002 was isolated from a forest disturbed by human activities, where the dominant species is *Juniperus deppeana*, located in Ixtacuixtla de Mariano Matamoros in the state of Tlaxcala, Mexico, molecularly identified with the ITS region and registered in the Microorganisms Collection of the National Center for Genetic Resources of INIFAP (CM-CNRG 700).

To propagate *A*. *pediades* mycelium, it was grown on agar plates with malt extract and incubated at 25 °C for 15 days. Agar cylinders were cut and stored in 15% glycerol at 4 °C, which were used for the following propagations.

For liquid cultures, *A*. *pediades* was grown in basal medium (BM) as previously reported [38], containing (g·L^−1^): glucose, 10; yeast extract, 5; KH_2_PO_4_, 0.6; MgSO_4_·7H_2_O, 0.5; K_2_HPO_4_, 0.4; FeSO_4_·7H_2_O, 0.05; MnSO_4_·H_2_O, 0.05; ZnSO_4_·7H_2_O, 0.001. Three conditions were used: basal medium (BM), basal medium with copper sulfate (BM+CuSO_4_ (0.019 mM)), and BM with hexachlorobenzene (BM+HCB (0.001 mM)). The cultures were performed in Erlenmeyer flasks containing 50 mL of medium, inoculated with one agar cylinder (with mycelium), and incubated at 27 °C and 115 rpm for 13 days. The first sampling was carried out after 72 h and subsequently every 48 h. The mycelium produced in each flask was filtered through filter paper Whatman^®^ no. 1, which also allowed obtaining the enzymatic extracellular crude extract (EECE). These extracts were stored in 50 mL tubes (Corning™ Falcon™) at −20 °C. The biomass was determined by dry weight, and the specific growth rate constant (*μ*) was obtained using the following formula:

(1) $$\mu =\frac{\ln B_{2}-\ln B_{1}}{t_{2}-t_{1}}$$

where *B*_1_ and *B*_2_ are the weights of the dry biomass for the cultivation times *t*_1_ and *t*_2_, respectively [39], which correspond to the extremes of the segment of the growth curve at which the maximum growth rate is presented.

### 2.2. Enzyme Activity and Zymogram Assay

The EECE were used to determine the extracellular laccase activity by spectrophotometry, monitoring the change in absorbance due to the oxidation of 2,6-dimethoxyphenol (2,6-DMP) using the JENWAY 6715 UV/Vis spectrophotometer (Cole Parmer, Vernon Hills, IL, USA). The standard reaction mixture (1 mL) contained 2,6-DMP (2 mM) in K_2_HPO_4_ (0.1 M, pH 6.5) and 40 µL of EECE. The reaction was monitored at 468 nm for 4 min at 39 °C [40,41]. The enzymatic activity was expressed in International Units (IU), where 1 IU was defined as the amount of enzyme that catalyzes the transformation of 1 µmol of 2,6-DMP into product per minute. The total protein concentration in the EECE was determined following Lowry et al. [42]. Furthermore, the EECE was used to determine the laccase activity by zymography in semi-denaturing SDS–PAGE in 11% acrylamide. The electrophoresis conditions were 150 V for 1–1.25 h. The zymogram was revealed using 2,6-DMP (2 mM) as substrate in K_2_HPO_4_ (0.1 M, pH 6.5). The semi-denaturing and denaturing SDS–PAGE gels were stained with colloidal Coomassie brilliant blue (R-250) (Sigma–Aldrich, Waltham, MA, USA).

### 2.3. Partial Purification of Laccase

To partially purify the laccase protein produced by *A. pediades*, the EECE obtained at 216 h of growth was concentrated using Millipore Amicon™ Ultra15 30,000 MWCO (Merck, Darmstadt, Germany) using centrifugation at 4 °C and 1700× *g* until 10% of the initial volume of EECE was obtained. Then, the concentrated EECE was loaded onto a Sephacryl 200 (GE Healthcare, Cheshire, UK) size exclusion column coupled to an Amersham Pharmacia Biotech AKTA FPLC system (GE Healthcare, Chicago, IL, USA). The column was previously equilibrated with equilibrium buffer K_2_HPO_4_ (50 mM, pH 7.35), and the proteins were eluted using the same equilibrium buffer at a flow rate of 0.5 mL/min. Subsequently, laccase activity was measured in each of the fractions using a standard reaction mixture. The laccase activity fractions were concentrated using Millipore Amicon ™ Ultra15 30,000 MWCO (Merck, Germany).

### 2.4. LC−MS/MS Analysis

The laccase protein band purified and visualized on an SDS–PAGE gel was extracted using a scalpel blade; the gel fragment containing the protein of interest was placed in an Eppendorf tube and resuspended in 100 µL of ddH_2_O. The sample was sequenced at the Institute of Biotechnology (UNAM, Mexico) using the following procedure. The sample was reduced with dithiothreitol (Sigma-Aldrich; St Louis, MO, USA), alkylated with iodoacetamide (Sigma-Aldrich) and digested “in gel” with Tripsin (Promega Sequencing Grade Modified Trypsin; Madison, WI, USA). For digestion of the sample with trypsin, an ammonium bicarbonate solution (50 mM, pH 8.2) was used, and the reaction was incubated for 18 h at 37 °C. Then, peptides produced by enzymatic cleavage were desalted with Zip Tip C18 (Millipore; Billerica, MA, USA) and applied in a Liquid Chromatography-Mass Spectrometry (LC-MS) system, coupled to an LTQ-Orbitrap Velos mass spectrometer (Thermo-Fisher Co., San José, CA, USA) with a nano-electrospray ionization source (ESI). 

### 2.5. Functional Characterization of Laccase Protein

#### 2.5.1. Determination of Native Status 

The native status was determined using gel filtration chromatography (GFC). The semi-purified protein was loaded onto a Sephacryl 200 (16/60) gel filtration column previously pre-equilibrated with K_2_HPO_4_ (50 mM, pH 7.35). The protein was eluted using the same equilibrium buffer at a flow rate of 0.3 mL/min, and the absorbance signal was monitored at 280 nm. Afterward, laccase activity was measured in each of the fractions using a standard reaction mixture. Gel filtration standards #151-1901 (Bio–Rad Laboratories, Hercules, CA, USA) were loaded on a Sephacryl 200 (16/60) gel filtration column using the same conditions for the laccase protein. The column was coupled to the Amersham Pharmacia Biotech AKTA FPLC system (GE Healthcare, Chicago, IL, USA) in both cases.

#### 2.5.2. Effect of pH and Temperature on Laccase Activity

The effect of pH on the activity of the laccase protein semi-purified from *A. pediades* was determined. The enzymatic activity was measured in a pH range from 3.0 to 8.0 using four different buffer systems: McIlvaine buffer (pH 3.0–6.0), 50 mM MES buffer (pH 6.0–6.75), 50 mM HEPES buffer (pH 6.75–8.0) and glycine (pH 9.0–10.0). The protein was incubated at each pH, and the enzymatic activity was monitored at 39 °C and 468 nm in a MULTISKAN GO spectrophotometer (Thermo Fisher Scientific, Waltham, MA, USA) using a standard reaction mixture. In the assay, the nonenzymatic reduction of 2,6-DMP was measured at each pH and subtracted from the experimental points. The pH stability of the purified laccase was assessed by preincubating the enzyme in the four different buffer systems mentioned above at 25 °C for 24 h. Then, the residual laccase activities were determined again with 2,6-DMP (2 mM) as the substrate [43,44,45,46].

The effect of temperature on laccase activity was determined by measuring laccase activity at different temperatures ranging from 4 °C to 70 °C and at the optimum pH using 2,6-DMP (2 mM) as a substrate. Residual enzyme activity was expressed as a percentage, and the highest activity was set to 100%. Finally, the thermostability of the laccase protein was determined by preincubating the protein adjusted to 7 µg in K_2_HPO_4_ (50 mM, pH 7.35) at different temperatures ranging from 20 to 80 °C for 20 min. Afterward, the laccase activity was determined as mentioned, at 39 °C. The residual activity was expressed as a percentage; the enzyme activity obtained at 20 °C was set to 100%. Thermal inactivation and pH assays were performed in triplicate.

#### 2.5.3. Determination of Steady-State Kinetic Parameters

Kinetic constant parameters of the laccase activity for 2,6-DMP substrates were studied. The steady-state kinetic parameters for the 2,6-DMP substrate were obtained from the initial velocity data by varying the concentration from 0 to 2 mM. The initial velocities obtained for each concentration were fitted to the Michaelis–Menten equation via nonlinear regression calculations [47], and the steady-state kinetic parameters *K*_m_, *k*_cat_, and *V*_max_ were obtained.

### 2.6. Statistical Analysis

Four experimental replicates were performed for the cultures of the fungus, while the gels and kinetic assays were performed in triplicate. All data were expressed as means ± standard deviations (SD) and were analyzed by one-way ANOVA to determine significant differences in the biomass produced and the specific laccase activity among treatments at each cultivation time. Two-dimensional ANOVA was used to determine the differences in the specific activity of laccase between culture times, followed by Tukey’s test. The results were considered significant when *p* was ≤0.05. The analyses were carried out with the XLSTAT Microsoft Excel 2011 complement.

## 3. Results

### 3.1. Effect of CuSO_4_ and HCB on the Growth and Laccase Activity of Agrocybe pediades

**Figure 1:**
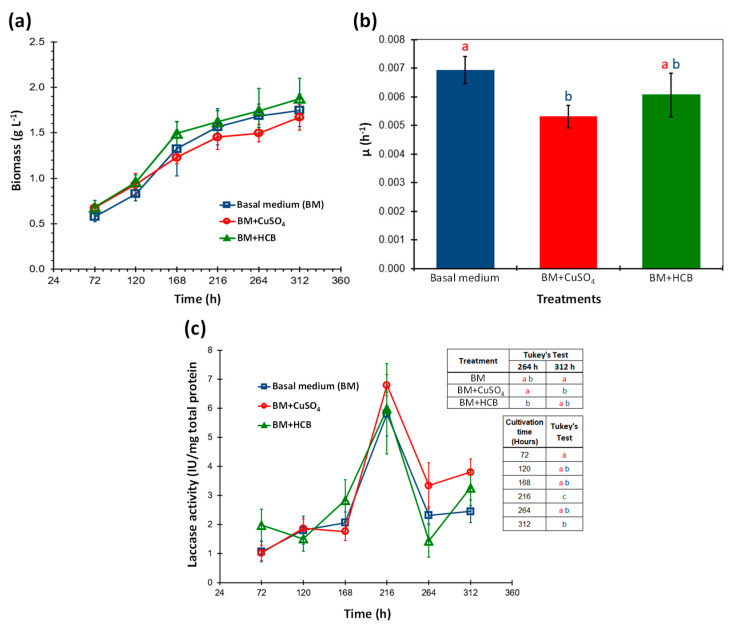
Growth and detection of extracellular laccase activity produced by *Agrocybe pediades* at different culture hours. (**a**) Biomass production. (**b**) Specific growth rate constant (µ) of the fungus. (**c**) Measurement of laccase activity. Basal medium (BM) (**□**); BM+CuSO_4_ (ο)_,_ and BM+HCB (Δ). The value shown for each time corresponds to the mean + standard deviation (SD). Different letters (a,b) indicate the statistical differences using Tukey’s test (*p* ≤ 0.05).

Liquid basal medium (BM) supplemented with copper (CuSO_4_) or hexachlorobenzene (HBC) were the treatments used to evaluate their effect on growth and extracellular laccase activity produced by *A*. *pediades*. The results showed (Figure 1a) that in all the treatments the same growth profile of the fungus was obtained with maximum biomass production at 312 h; no significant differences were observed between them according to the ANOVA and F test for each time point (F = 4.25, 2.04, 1.96, 1.22, 2.04, 2.01 < F_α(2,9)_ = 4.26, *p* = 0.05). 

When the specific growth rate constant (*μ*) of each treatment was calculated, it was found that the value of (*μ*) of the medium in the presence of copper was lower compared to the other treatments, indicating that copper has a slight effect on the development of the fungus. Statistical analyses by ANOVA and F-test (F = 8.27 > F_α(2.9)_ = 4.26) showed that the value (*μ*) of the control (BM) was significantly different from the copper medium, but not from the HCB-containing medium, which was confirmed and visualized with the Tukey’s test (Figure 1b). 

Similarly, the laccase activity was determined in the EECE of each of the treatments at the same culture times. The activity was reported in IU with respect to the amount of total protein secreted in the cultures. Extracellular laccase activity was detected in all treatments (Figure 1c), and no significant differences in enzymatic activity among treatments for each culture time (72, 120, 168 and 216 h) were observed using ANOVA and F test for each time point (F = 2.17, 0.41, 1.43, 0.27 < F_α(2,9)_ = 4.26, α = 0.05), except for 264 (H = 13.95 > χ^2^_α(2)_ = 5.991, α = 0.05) and 312 h (F = 7.8337 > F_α(2,9)_ = 4.26, α = 0.05). The highest laccase activity was obtained at 216 h of culture in all treatments (6.25 IU/mg on average), and this time was statistically different from the rest of those analyzed, according to ANOVA and the F test (F = 31.34 > F_α(5,54)_ = 2.40, *p* = 0.05)**.** While the mean comparison analysis (Tukey’s test) performed for times 264 and 312 h showed that in the BM+CuSO_4_ treatment, laccase activity was higher and significantly different from the activity recorded for the control and BM+HCB (Figure 1c, inset table). Our data indicated that laccase activity is not proportional to the biomass produced and neither is it favored by the presence of CuSO_4_.

### 3.2. Analysis of Enzymatic Activity by Zymogram

**Figure 2:**
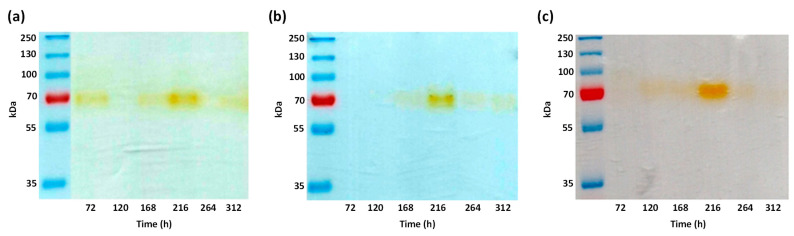
Detection of laccase activity by zymography assays from enzymatic extracellular crude extract (EECE). (**a**) Control (BM); (**b**) BM+CuSO_4_; and (**c**) BM+HCB. 2,6-Dimethoxyphenol (2,6-DMP) was used as a substrate at a concentration of 2 mM and 7 µg of total protein was loaded into each lane. Brown band indicates positive reaction to laccase.

To determine the presence of the protein (and possible isoenzymes) with laccase activity in the EECE produced by *A*. *pediades* in liquid medium, zymography analysis was performed for each of the three treatments. Semi-denaturing protein SDS–PAGE electrophoresis was performed on the extracts of each treatment and culture time (Figure 2). In the gels obtained from the three treatments, only one band with a molecular weight (MW) of approximately 70 kDa was detected (brown band), indicating that the addition of CuSO_4_ (Figure 2b) or HCB (Figure 2c) at the concentrations used in the cultures did not induce the expression of other isoenzymes with laccase activity. Since no differences were observed between the zymograms of the control and the possible inducers (copper or HCB), only BM was used for the purification and characterization of the protein produced by *A*. *pediades*.

### 3.3. Partial Purification of Laccase Protein

**Figure 3:**
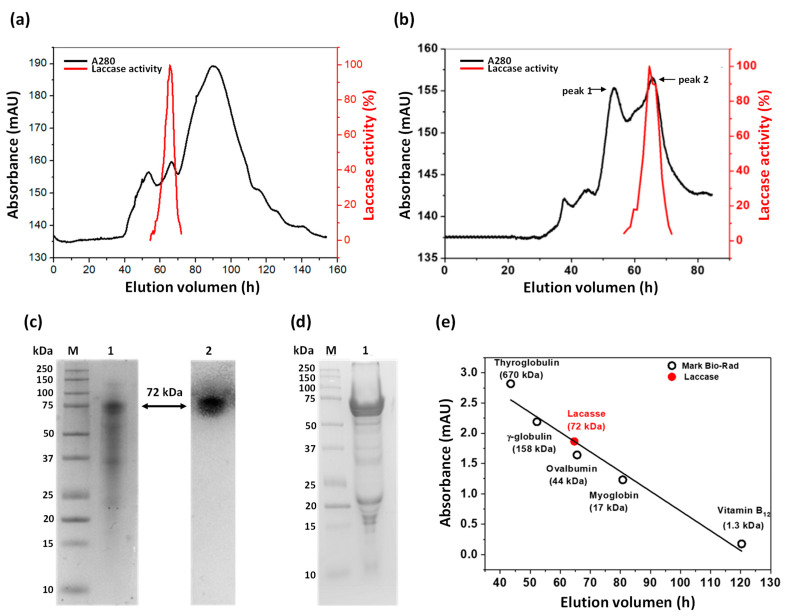
Purification of laccase enzyme produced by *A*. *pediades*. (**a**,**b**) Chromatogram of the proteins separated by the Sephacryl 200 exclusion column (black line). Laccase activity of the fractions (red line). (**c**) Semi-denaturing SDS–PAGE of the purified laccase enzyme. Lane 1: Purified laccase enzyme. Lane 2: Confirmation of laccase production by zymogram analysis using 2,6-DMP (2 mM) as substrate; 40 µg of total protein was loaded into each lane. (**d**) Denaturing SDS–PAGE gel. Lane 1: Purified laccase enzyme incubated with β-mercaptoethanol (10 µg of total protein). M: Protein molecular weight (MW) marker precision plus protein kaleidoscope. (**e**) Calibration plot showing the elution volumes of the Bio–Rad gel filtration standard (black spots) versus the log of the molecular weight (MW) of the laccase protein (red spot).

The laccase enzyme was partially purified from the concentrated EECE obtained at 216 h of culture. Figure 3a (black line) shows the elution profile of the proteins present in the EECE using Sephacryl 200 (16/60) gel filtration column. The laccase activity was measured in each of the fractions, and the fractions corresponding to the second protein peak (from 55 to 65 mL elution) showed laccase activity (Figure 3a, red line). In order to improve the purification and subsequent determination of the native state of the protein, the previously obtained fractions were again subjected to the gel filtration column. These fractions were again separated into two peaks, the first with an elution volume of 53 mL but without enzymatic activity, while the second peak (eluted at 65 mL) was the one that showed laccase activity (Figure 3b). The presence of a peak with activity in the EECE coincided with the only band with laccase activity observed at 216 h of the zymography assays (Figure 2). 

A summary of the laccase purification is shown in Table 1. A total protein of 1.3 mg with a specific activity of 285 µmol∙min^−1^∙mg^−1^ and a yield of 8.7% was obtained.

To corroborate the purity of the purified laccase protein, a semi-denaturing SDS–PAGE gel was performed, where a band of 72 kDa of greater intensity was visualized (Figure 3c, lane 1). Additionally, two bands of lower intensity and molecular weights (MW) of 35 kDa and 22 kDa were also observed. However, when the SDS-PAGE gel was incubated with 2,6-DMP, only the band corresponding to 72 kDa was the one that reacted with the substrate (Figure 3c, lane 2). This data is consistent with the laccase activity that was reported in the previous zymograms (Figure 2), where also only one band was observed. Nevertheless, when treating the laccase protein with β-mercaptoethanol and heating it, and then running it on a denaturing SDS-PAGE gel, a band of greater intensity was observed with MW between 55 and 60 kDa (Figure 3d), which may be due to its possible deglycosylation.

### 3.4. Functional Characterization of Laccase Protein

#### 3.4.1. Determination of Native Status

The fractions that presented laccase activity shown in Figure 3b (red line), were collected and concentrated to determine the oligomeric state of the protein. For this, a Sephacryl 200 column was used, which was previously calibrated with known standards (Bio–Rad). According to the calculations, the molecular weight obtained for the extracellular laccase protein of *A*. *pediades* is 72 kDa (Figure 3e).

This result agrees with that previously obtained in semi-denaturing SDS–PAGE, where a band of 72 kDa was detected by zymography (Figure 2c, lane 2), indicating that the extracellular laccase protein purified from the EECE of *A. pediades* culture in the native form is a monomer.

**Figure 4:**
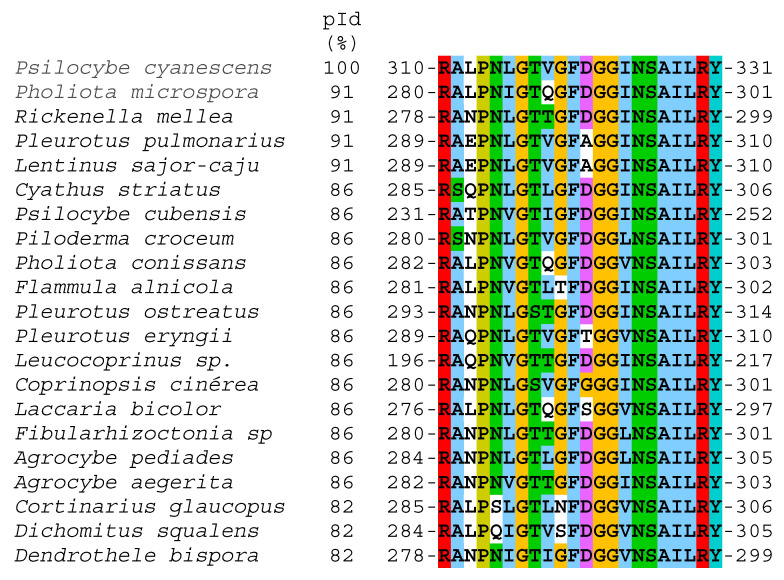
Multiple alignment of the partial amino acid sequence obtained from *A. pediades* (strain CIGyA-002) and other fungal laccases. The species and their respective accession numbers were *Psilocybe cyanescens* (PPQ94072.1), *Pholiota microspora* (BAU94251.1), *Rickenella mellea* (TDL19075.1), *Pleurotus pulmonarius* (KAF4569369.1), *Lentinus sajor-caju* (CAD45378.1), *Cyathus striatus* (KAF8994970.1), *Psilocybe cubensis* (KAH9477711.1), *Piloderma croceum* (KIM83705.1), *Pholiota conissans* (KAF9477937.1), *Flammula alnicola* (KAF8959650.1), *Pleurotus ostreatus* (AAR21094.1), *Pleurotus eryngii* (KAF9493880.1), *Leucocoprinus* sp. (AFV15795.1), *Cortinarius glaucopus* (KAF8805505.1), *Agrocybe pediades* (KAF9550055.1), *Coprinopsis cinérea* (AAD30966.1), *Laccaria bicolor* (XP_001886681.1), *Agrocybe aegerita* (CAA7260498.1), *Fibularhizoctonia* sp. (KZP20931.1), *Dichomitus squalens* (TBU48751.1), *Dendrothele bispora* (THU97492.1). Alignment was performed using ClustalW and visualized with Jalview. Solid (uniform) colors indicate conserved amino acids and non-conserved amino acids without color.

The purified protein was analyzed by LC-MS/MS, several partial amino acid (aa) sequences were generated; among these, a peptide was identified as RALPNLGTVGFDGGINSAILRY. When searching for these amino acids in the BLASTp (NCBI) database, the peptide was located in sequences of proteins that contain the cupredoxin domain (containing type I copper centers) and that coincide with blue copper proteins in the included laccases (Figure 4). The alignment of this amino acid sequence shows high identity with different fungal laccases such as *Psilocybe cyanescens* (100%) and *A. pediades* (86%), among others (Figure 4).

#### 3.4.2. Effect of pH and Temperature on Laccase Activity

**Figure 5:**
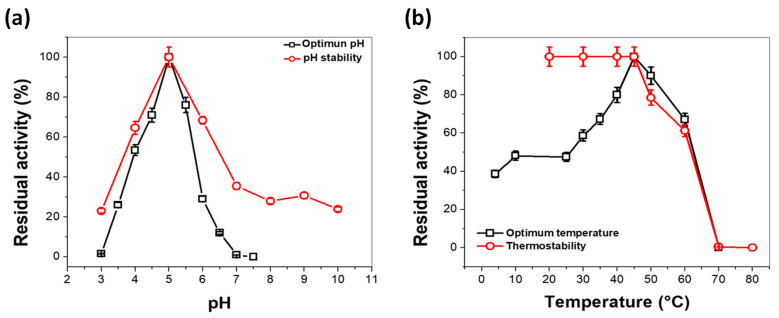
Determination of optimum pH and thermostability for the activity of the laccase protein purified from *A. pediades*. (**a**) Evaluation of laccase activity at different pH (black boxes) and stability (red circles). (**b**) Effect of temperature on the activity (black box) and stability (red circle). Error bars indicate the mean ± standard deviation of the triplicate values.

The optimal pH for laccase from *A. pediades* was determined by measuring its activity over a range of pH from 3.0 to 7.5. More than 50% of the enzymatic activity was obtained from pH 4.0, and 100% of the activity was found at pH 5.0, after pH 6.0 the activity decreases drastically (Figure 5a black squares). Based on the pH profile, the extracellular laccase enzyme from *A. pediades* has an optimum pH of 5.0. Therefore, the following assays were performed at pH 5.0. Regarding the pH stability profile, the laccase from *A. pediades* was stable in a range of pH values from 4.0 to 6.0, maintaining more than 60% of its original activity after incubation at 25 °C for 24 h (Figure 5a, red circle). 

The optimum pH obtained for the laccase of *A. pediades* is similar to the optimum pH values of laccase previously reported (Table 2). 

While the optimum temperature was determined to be in the range of 4–70 °C. The laccase of *A. pediades* showed its maximal activity at 45 °C, and a high level of maximal activity (>50%) was detected, ranging from 30 °C to 60 °C. In comparison, at 60 °C, the activity was dramatically reduced (Figure 5b). Temperature stability regarding laccase activity was also determined (Figure 5b). The results showed that the activity remained intact from 20 °C to 45 °C and slowly decreased at 50 °C after the enzyme was incubated at test temperatures for 20 min. Subsequently, the activity of the enzyme decreased rapidly from 50 °C until total loss of activity at 70 °C. Furthermore, we observed that at 62 °C, the enzyme loses 50% of its activity (T_50_), indicating that this protein is stable at high temperatures.

#### 3.4.3. Determination of Steady-State Kinetic Parameters

**Figure 6:**
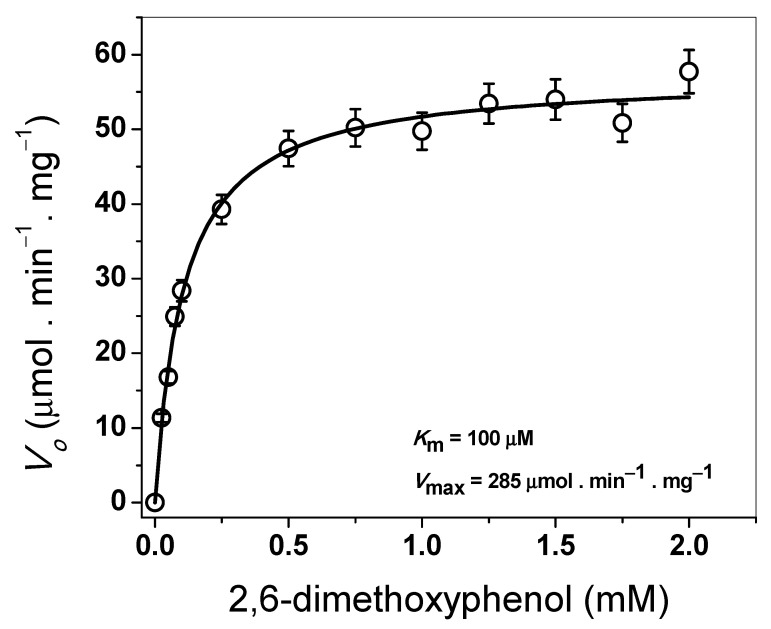
Determination of steady-state kinetic parameters of extracellular laccase purified from *A*. *pediades* cultures. Initial velocity data for the 2,6-DMP substrate were obtained for varying substrate concentrations, indicated in the abscissa axis, and fitted to the Michaelis–Menten equation by nonlinear regression calculations.

The steady-state kinetic parameters were determined spectrophotometrically by varying the concentration of the 2,6-DMP substrate from 0 to 2 mM. Figure 6 shows the hyperbolic behavior for the 2,6-DMP substrate when measuring the laccase activity.

The apparent *K*_m_ value for 2,6-DMP was 100 µM, with an apparent *V*_max_ of 285 µmol·min^−1^·mg^−1^ (Figure 6). According to these results, the extracellular laccase produced by *A. pediades* has a five-fold higher affinity for the substrate 2,6-DMP than the laccases produced by fungi of other species (Table 2). A *k*_cat_ value of 342 s^−1^ was determined for the laccase of *A. pediades*.

#### 3.4.4. Effect of DL-Dithiothreitol (DTT) on Laccase Activity

**Figure 7:**
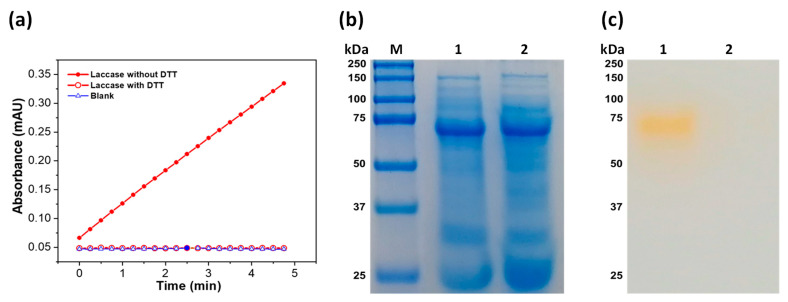
Effect of DTT on the enzymatic activity of laccase from *A. pediades*. (**a**) The catalytic activity of laccase in the presence and absence of DTT. (**b**) 12%-SDS–PAGE of laccase in the presence (lane 2) and absence of DTT (lane 1). The laccase protein was incubated with DTT (15 mM) for 5 min. (**c**) Zymogram of laccase with (lane 2) and without DTT (lane 1). In each lane, 20 µg of laccase protein was loaded. Zymography was carried out using 2,6-DMP (2 mM) as a substrate.

Finally, to determine whether the enzyme contains disulfide bridges in its structure, the effect of DTT on the enzymatic activity of purified laccase from *A. pediades* was evaluated. The protein was incubated in the presence and absence of DTT, and the catalytic activity was measured. As seen in Figure 7a, in the presence of DTT, the laccase enzyme loses 100% of its activity compared to the absence of DTT. In addition, the protein incubated under the same conditions was analyzed by SDS–PAGE, and the same protein profile was observed in both the presence and absence of DTT (Figure 7b). Regarding enzyme activity on SDS–PAGE, we observed a band that gave a positive reaction with the 2,6-DMP substrate in the absence of DTT, whereas in the presence of DTT, no activity was observed (Figure 7c).

## 4. Discussion

WRFs play an essential role in the environment because they have the capacity to process the three components of lignocellulosic biomass (cellulose, hemicellulose and lignin) [3]. These fungi can use lignin as the only source of carbon and energy and mineralize it to CO_2_ [28]. This action is due to the LMEs that they produce, such as lignin peroxidase, manganese peroxidase and laccase [5]. Moreover, these LMEs have become more relevant due to their ability to degrade different pollutants present in agro-industrial residues or wastewater mainly from agricultural and industrial activities [7]. However, laccase stands out from peroxidase because it only uses oxygen from the air as its only cofactor and releases water as a byproduct; for this reason laccase is considered a “green tool” [10,28]. In view of this, various studies have reported that fungi are an important source of laccase production with different biochemical properties, and that when testing their enzymatic extracts or free enzyme they have shown high efficiencies in the biotransformation of different contaminants.

In this work, a saprophytic fungus isolated from a *J. deppeana* forest was used. This forest has been severely affected by various anthropogenic activities, mainly by fires for agricultural use, which also leads to the use of different herbicides. These activities have caused changes to the structure, dynamics and species present in that area. Interestingly, this fungus is one of the few species that continues to grow in this forest, and which was identified by morphological characteristics and molecularly (using the ITS region) as *Agrocybe pediades* (unpublished). Therefore, it was of interest to evaluate its development in vitro and, in particular, the production of the extracellular laccase enzyme. For this, *A*. *pediades* was grown in liquid medium previously used for laccase production in *P*. *ostreatus* [38].

As is known, Cu^2+^ plays a key role as a metal activator of the laccase active site and can induce both transcription and its activity [10,33]. This metal ion has been used as an inducer at different concentrations and has considerably improved the expression of laccases in a range of 3.4, 3.6, 4 and 160-fold in the fungal species *Coprinus comatus* [32], *P. eryngii* [23], *P. sajor-caju* [8] and *Hexagonia hirta* [35], respectively. Initially, the medium used to grow *A. pediades* contained CuSO_4_ (1.5 mM), but at this concentration its growth was strongly inhibited (data not shown), so it was necessary to use a low concentration (0.019 mM), which still showed a slight effect on the specific growth rate (*μ*). Regarding the enzymatic activity, with that concentration of Cu^2+^, the laccase activity in BM+CuSO_4_ was higher (1.0-fold) with respect to the control (BM), a result that shows less dependence on Cu^2+^ compared to other fungi. Sajben-Nagy et al. [17] also observed a lower induction of CuSO_4_ on the laccase activity of the fungus *Leptosphaerulina chartarum* over time, when a 0.012 mM concentration was used, similar to the concentration used and results obtained in this study.

Further, fungal laccases have been documented to oxidize a wide range of substrates, including aromatic compounds [5,7,28]. For this reason, several researchers implemented the use of these compounds in synthetic medium or took advantage of those present in industrial waste to increase laccase production [7]. HCB is an aromatic compound used as a fungicide and generated as a byproduct and residue during the manufacture of bleached paper pulp. Hence, we used HCB in order to evaluate its effect as an inducer for the production of laccase in *A. pediades*. Although HCB did not improve enzyme production, it is important to note that *A. pediades* demonstrated its ability to tolerate and grow in the presence of this toxic compound. Therefore, the fungus and its laccases can be used to perform degradation studies on this compound and other xenobiotics.

On the other hand, in the zymography tests only one band was observed, with constant presence in the three treatments and at all times evaluated (Figure 2). This result rules out that HCB or CuSO_4_ induces new laccase isoenzymes or potentiates their activity in this fungus, unlike what has been reported for ligninolytic fungi *P. eryngii*, where laccase activity is increased in the presence of 1.0 mM copper [23]. 

According to the complete genome sequencing of *A. pediades* strain AH40210 (www.ncbi.nlm.nih.gov/bioproject/?term=PRJNA410408; accessed on 20 August 2021), there are at least nine laccase enzymes (GenBank accession: KAF9550055.1, KAF9554181.1, KAF9555437.1, KAF9558355.1, KAF9558364.1, KAF9559431.1, KAF9563577.1, KAF9563579.1, KAF9563865.1). Nevertheless, under the conditions tested in this work, only the activity of one isoenzyme was consistently detected in all treatments during 13 days of culture. These data and those recently reported by Aza et al. [51], show that the physiology and secretion of laccase of *A. pediades* changes radically when it is cultured in liquid and solid. These authors only found one laccase (ID (JGI): 639182) in non-ligninolytic conditions (liquid medium), up to 43 days, without constant expression; while under ligninolytic conditions (solid medium) they also only detected one laccase (ID (JGI): 823363) on days 6 and 14 of culture, which was precisely the one that coincided with the peptide sequenced in our work with 86% identity. 

The enzyme was partially purified from the EECE of *A. pediades* control culture. The yield of 8.7% obtained agrees with that previously reported by Wu et al. [15], where a 9% yield was obtained using the same methodology for the purification of extracellular laccase from *F. solani*, whereas Chen et al. [16] had a lower yield (7.49%) in the purification of extracellular laccase from *Thermobifida fusca*. After purification, the protein was analyzed in SDS-PAGE and zymogram gels, and only a band with laccase activity was observed of approximately 70 kDa, consistent with the band observed in the extracellular extracts (Figure 2a,c) and very similar to the extracellular laccase also detected by zymography of the fungus *P. chrysogenum* [22]. The MW of laccase (72 kDa) from *A. pediades*, in its native state, was similar to the MW of the fungal laccases reported for *L. edodes* [14], *P. ostreatus* [31] and *Thielavia* sp. [21]. This was slightly higher than those reported for the laccases of various fungi belonging to the order Agaricales [48,49,50] and the previously purified laccase of *Stropharia aeruginosa*, with a laccase of 55 kDa [52]. Laccases differ in sequence length between species, but another variation in MWs may be due to their glycosylation. The carbohydrate content for this post-translational modification in laccases can be 10–45% and have three to ten potential glycosylation sites depending on the presence of the Asn-X-Thr/Ser consensus sequence [24]. The MW (72 kDa) of the protein is possibly due to the glycosylation it presents, which was confirmed by Aza et al. [51] when visualizing the laccase of *A. pediades* by zymograms using 2,6-DMP. They detected bands in the gel of 100 to 250 kDa, and after treating the protein with Endoglycosidase H, the laccase resulted in a final MW of 55 kDa, indicating its high degree of glycosylation.

Some enzymatic properties were determined, the optimum pH for the laccase of *A. pediades* was 5.0, very similar to that previously reported for laccases from *Trametes orientalis* [46], where the pH stability profile indicated that it was stable within pH 4.0–5.0, retaining more than 80% of its original activity. Further, it is interesting that laccases are stable and functional at acidic or neutral pH values but lose their activities under alkaline conditions [53].

While the optimum temperature for laccase activity was 45 °C, this value of temperature is consistent with the reported laccases of *T. pubescens*, *T. hirsute*, and *Ganoderma austral*, where maximal activity at temperatures ranging from 25 °C to 70 °C was observed [43,44,46]. The laccase enzyme from *A. pediades* was stable at high temperatures by showing a T_50_ at 62 °C. This effect of the temperature determined for the laccase agrees with that previously reported by Aza et al. [51] for the 7F12 laccase and NGly variants, where T_50_ (10 min) values of 59 °C were observed. In addition, this result agrees with that previously reported by Daroch et al. [52] in a glycosylated yellow laccase produced by *Stropharia aeruginosa*, which loses its activity at 70 °C, and a T_50_ of 62 °C was determined, which is consistent as both enzymes of *S. aeruginosa* and *A. pediades* belong to the same family. The comparison of these parameters with that reported in other fungi was summarized in Table 2.

Regarding the kinetic parameters *K_m_* (100 µM) and *V_max_* (285 µmol·min^−1^·mg^−1^), the extracellular laccase produced by *A. pediades* had a five-fold higher affinity for the substrate 2,6-DMP than the laccases produced by fungi of other species (Table 2). While the *k*_cat_ value obtained (342 s^−1^) is congruent with that previously reported for the laccase of *G*. *australe*, where a *k*_cat_ value of 237 s^−1^ was reported with 2,6-DMP as a substrate [44]. Other *k*_cat_ values ranging from 129 s^−1^ to 399 s^−1^ were obtained using ABTS as substrates in laccases of *T. hirsute*, *Cerrena unicolor GSM-01*, and *T. trogii BAFC 463*, among others [19,20,46]. *Km* values suggest that the laccase enzyme purified from *A. pediades* could perform more efficient catalysis on certain compounds than other enzymes, as previously observed [36], where the laccase enzymes that presented higher affinity for 2,6-DMP also had a similar affinity for sinapic acid and the syringaldazine compound.

Finally, it was observed that DTT acts as a laccase inhibitor, because when it was used a complete loss of its activity was recorded, which suggests the presence of disulfide bridges in the laccase protein or the presence of copper at the active site that is coordinated with an oxidized cysteine, and when DTT is added, cysteine is reduced, causing a loss of activity, as previously reported by Dwivedi et al. [53].

## 5. Conclusions

Here, we partially purified and identified an extracellular laccase (laccI) of *A*. *pediades* from liquid culture. We note that the addition of CuSO_4_ or HCB to the cultures does not induce the production of isoenzymes in *A*. *pediades* compared to that obtained with the basal medium. A constant laccase was purified from the EECE of *A. pediades*; the purified protein was obtained as an active monomer with an approximate molecular mass of 55 to 60 kD and 72 kDa in its native state. The increase in resistance to temperature (T_50_ = 62 °C) suggests that this protein is stable at high temperatures. The kinetic parameters of the laccase enzyme revealed higher *V*_max_ and *K*_m_ values than those of laccases from other fungi, showing outstanding catalytic capabilities on 2,6-DMP substrate. This work could serve as a reference for further research on other substrates that this enzyme can catalyze, as well as testing this laccase on toxic compounds.

## Figures and Tables

**Table 1:** Purification of extracellular laccase from *A. pediades*.

Purification Steps	Total Protein (mg)	Specific Activity (µmol∙min^−1^∙mg^−1^)	Total Activity (µmol∙min^−1^)	Yield (%)
EECE	1518	2.8	4250	100
Sephacryl 200	1.3	285	370.5	8.7

**Table 2:** Properties of laccase enzymes purified from fungi of the Agaricales order.

Family	Species	Molecular Weight (kDa)	pH	Temperature (°C)	Km (μM)	Reference
Strophariaceae	*Agrocybe pediades*	72	5.0	40	100	This work
Agaricaceae	*Agaricus blazei*	66	5.5	n.d *	1026	[48]
Marasmiaceae	*Lentinula edodes*	72	4.0	40	557	[14]
Marasmiaceae	*Moniliophthora perniciosa*	57	6.5	55	n.d.	[49]
Pleurotaceae	*Pleurotus ostreatus*	83−85	5.5	35	8800	[31]
Pluteaceae	*Volvariella volvacea*	58	4.6	45	570	[50]
Chaetomiaceae	*Thielavia* sp.	70	5.0	70	24	[21]

* n.d.: not determined.

## Data Availability

The data presented in this study are contained within this article.
